# Severe COVID-19 and aging: are monocytes the key?

**DOI:** 10.1007/s11357-020-00213-0

**Published:** 2020-06-15

**Authors:** Brandt D. Pence

**Affiliations:** 1 School of Health Studies, University of Memphis, Memphis, TN 38152 USA; 2 Center for Nutraceutical and Dietary Supplement Research, University of Memphis, Memphis, TN 38152 USA; 3 University of Memphis, 304 Elma Roane Fieldhouse, 495 Zach H. Curlin St., Memphis, TN 38152 USA

**Keywords:** SARS-CoV-2, Monocyte, Macrophage, Inflammaging, Cytokine storm, Aging

## Abstract

The ongoing pandemic severe acute respiratory syndrome coronavirus-2 (SARS-CoV-2) causes a disproportionate number of severe cases and deaths in older adults. Severe SARS-CoV-2-associated disease (coronavirus disease 2019 (COVID-19)) was declared a pandemic by the World Health Organization in March 2020 and is characterized by cytokine storm, acute respiratory distress syndrome, and in some cases by systemic inflammation–related pathology. Currently, our knowledge of the determinants of severe COVID-19 is primarily observational. Here, I review emerging evidence to argue that monocytes, a circulating innate immune cell, are principal players in cytokine storm and associated pathologies in COVID-19. I also describe changes in monocyte function and phenotype that are characteristic of both aging and severe COVID-19, which suggests a potential mechanism underlying increased morbidity and mortality due to SARS-CoV-2 infection in older adults. The innate immune system is therefore a potentially important target for therapeutic treatment of COVID-19, but experimental studies are needed, and SARS-CoV-2 presents unique challenges for pre-clinical and mechanistic studies in vivo. The immediate establishment of colonies of SARS-CoV-2-susceptible animal models for aging studies, as well as strong collaborative efforts in the geroscience community, will be required in order to develop the therapies needed to combat severe COVID-19 in older adult populations.

## Introduction

In late 2019, a viral pneumonia of unknown etiology caused an epidemic in Hubei Province, China (Huang et al. 2020; Zhou et al. 2020b). Early cases were linked to a seafood market in Wuhan, and as the location and time of year in which the virus emerged were similar to the 2002–2003 severe acute respiratory syndrome coronavirus (SARS-CoV-1) epidemic, samples from several patients were tested for coronavirus infections. Analysis of the viral genome identified a coronavirus with approximately 80% sequence identity with SARS-CoV-1 and which closely matched a previously identified bat coronavirus (Zhou et al. 2020b). The virus, initially named novel coronavirus 2019 (2019-nCoV) and subsequently reclassified as SARS-CoV-2 (Gorbalenya et al. 2020), quickly spread beyond the borders of China and was declared pandemic by the World Health Organization on 11 March 2020 (Ghebreyesus 2020). As of 21 May 2020, the virus-associated disease now called coronavirus disease 2019 (COVID-19) has resulted in over 5 million confirmed cases and over 330,000 deaths worldwide (Worldometer 2020).

## Severe COVID-19 and pathogenesis of SARS-CoV-2

SARS-CoV-2 is capable of person-to-person transmission (Phan et al. 2020; Chan et al. 2020b) and can persist in aerosols and on surfaces for periods of at least several hours (van Doremalen et al. 2020). Transmission is thought to occur primarily through respiratory droplets and direct contact with contaminated surfaces (Desai and Patel 2020), although fecal-oral routes of spread are also suspected (Xiao et al. 2020). COVID-19 presents with typical symptoms of fever, dry cough, myalgia, shortness of breath, and pneumonia (Huang et al. 2020; Chen et al. 2020b), and patients with severe COVID-19 often develop acute respiratory distress syndrome (ARDS). Systemic complications are less common but can include pathology in the cardiovascular (Zheng et al. 2020), renal (Ronco and Reis 2020), hepatic (Zhang et al. 2020a), gastrointestinal (Yeo et al. 2020), and central nervous (Baig et al. 2020) systems. Recent evidence suggests that asymptomatic and pre-symptomatic individuals are capable of infecting others (He et al. 2020), possibly due to viral shedding from the upper respiratory tract during the pre-symptomatic period (Wölfel et al. 2020). The evolving nature of our understanding of viral dynamics and COVID-19 symptom presentation has caused substantial variability in estimations of infection-fatality rates, percentage of individuals who develop severe disease, and other population-level metrics, and attempts to calculate these are often outdated or superseded within days. However, SARS-CoV-2 is estimated to be at least an order of magnitude more deadly than seasonal influenza virus (Faust and del Rio 2020).

A principal determinant of severe COVID-19 appears to be a pronounced increase in systemic pro-inflammatory cytokines and other inflammatory markers (Chen et al. 2020a; Huang et al. 2020; Qin et al. 2020), indicative of a cytokine storm corresponding to that seen in other viral infections and bacterial sepsis (Chousterman et al. 2017; Teijaro 2017). Clinical markers characteristic of cytokine storm include increased circulating C-reactive protein and erythrocyte sedimentation rate (Zhang et al. 2020d), and observational studies have noted upregulation of a variety of circulating cytokines in COVID-19 (Schett et al. 2020; Zhou et al. 2020d). Among these, interleukin (IL)-6 has received particular attention, and clinical trials on IL-6 receptor antagonists and other biologics are underway (Mahase 2020; Schett et al. 2020). It is thought that cytokine storm mediates many of the systemic effects in severe COVID-19, although viremia may also play a role, as viral RNA has been found in fecal samples and blood (Young et al. 2020; Guo et al. 2020b), and a variety of tissues express high levels of the principal viral receptor, angiotensin-converting enzyme-2 (ACE2) (Qi et al. 2020) and thus may be permissive to viral infection and replication. Indeed, a recent study of autopsy samples from 27 patients with fatal COVID-19 reports widely disseminated virus detectable in the brain, heart, kidneys, and other organs (Puelles et al. 2020), demonstrating the potential for SARS-CoV-2 to directly cause pathogenesis outside the pulmonary system.

## Aging and COVID-19

Early in the SARS-CoV-2 outbreak, it was noted that older adults accounted for a disproportionate number of severe cases and deaths due to COVID-19 (Wang et al. 2020b), and this has been corroborated by a number of epidemiological and observational studies (Nikolich-Žugich et al. 2020; Onder et al. 2020; Ruan et al. 2020; Shahid et al. 2020; Wu et al. 2020; Zhou et al. 2020a; Wang et al. 2020c). Advanced age is now considered the principal risk factor for COVID-19 complications. As dysregulations of immune function, vaccine responses, and inflammation in older adults are well-known and have been extensively reviewed (Pinti et al. 2016; Ciabattini et al. 2018; Nikolich-Žugich 2018), it has been speculated that immunosenescence is a key determinant of outcomes in SARS-CoV-2 infections (Nikolich-Žugich et al. 2020). However, the relative impact of immune system dysregulation on pathogenicity during COVID-19 in older adults (or indeed in anyone) is not yet well-understood. In the remainder of this review, I compare emerging knowledge on innate immune responses in severe COVID-19 to known aspects of age-related immune dysregulation, in order to argue for a principal contribution of circulating monocytes to COVID-19 severity in older adults.

To date, a small number of studies have examined immune system function as regulated by SARS-CoV-2. Because of the often-substantial publication delay due to peer review of manuscripts, many of the studies covered here are still in preprint stage. As this is an emergent situation with substantial public health implications, I have included these in the interest of providing the fullest and most up-to-date information possible. However, the reader should note that some conclusions herein are based on papers which have not yet been certified by peer review and formal publication.

## Contributions of monocytes and macrophages to severe COVID-19

Monocytes are cells of the innate immune system (Jakubzick et al. 2017) that participate in inflammatory responses, phagocytosis, antigen presentation, and a variety of other immune function processes. Circulating monocytes also extravasate into peripheral tissues to differentiate into macrophages or dendritic cells during sterile and non-sterile inflammation. In humans, monocytes are generally described as consisting of three subsets based on their relative expression of CD14 and CD16; these are commonly identified as classical (CD14^+^CD16^−^), intermediate (CD14^+^CD16^+^), and non-classical (CD14^dim^CD16^+^) subtypes (Ziegler-Heitbrock et al. 2010). However, recent efforts using single-cell technologies have characterized additional phenotypes based on gene and protein expression patterns (Villani et al. 2017; Hamers et al. 2019; Wolf et al. 2019). The relative proportions of these subtypes are associated with a variety of diseases, with increasing severity generally linked to increased prevalence of the CD16^+^ intermediate and non-classical subsets (Kapellos et al. 2019; Narasimhan et al. 2019).

Several observational studies have characterized monocytes during SARS-CoV-2 infection. Zhou et al. (Zhou et al. 2020c) found significantly increased circulating proportions of CD14^+^CD16^+^ monocytes in peripheral blood from 33 hospitalized patients with diagnosed COVID-19, and this percentage was substantially increased in COVID-19 patients with ARDS. These monocytes appeared (by CD14 and CD16 staining) to be primarily of the intermediate subtype. Intermediate monocytes make up approximately 5% of the total monocyte population in healthy individuals, but in patients with severe COVID-19, this proportion increased to over 45%. Additionally, a significant increase in monocytes producing IL-6 was noted in mild COVID-19 patients (*N* = 21), and this was increased further in patients with severe disease (*N* = 12), suggesting that monocytes are key contributors to cytokine storm in COVID-19.

Likewise, a population of vacuolated monocytes with unusual forward scatter characteristics has been identified by flow cytometric analysis of blood collected from 28 COVID-19 patients (Zhang et al. 2020b). These cells were CD14^+^CD16^+^ and expressed a variety of macrophage markers, suggesting some degree of differentiation of monocytes to macrophages in the circulation during SARS-CoV-2 infection. This population of circulating monocytes/macrophages also expressed high levels of cytokines such as IL-6, TNFα, and IL-10, suggesting a contribution of these cells to cytokine storm, and increased proportions of these cells were associated with increased ICU admissions and prolonged time to hospital discharge. The total monocyte population was highly enriched for intermediate and non-classical subtypes in COVID-19 patients in the same study (Zhang et al. 2020b). However, as these findings were not compared with a healthy control group, some caution is necessary in interpreting the results from this study. Based on our existing knowledge of monocyte biology, it is likely that these patterns of monocyte activation are abnormal and associated with disease, but this was not confirmed experimentally in the referenced study.

Likewise, a large cross-sectional study profiled immune cells in the circulation and lungs of patients with mild (*N* = 19), severe (*N* = 21), and critical (*N* = 24) COVID-19, as well as healthy donors (*N* = 22) (Sanchez-Cerrillo et al. 2020). Supporting the above, the authors found a substantial decrease in monocytes in the circulation in COVID-19 patients. This was coupled with a severity-specific enrichment of intermediate and non-classical monocytes in the lungs of patients with severe and critical disease, suggesting an association between pro-inflammatory monocyte trafficking to the lungs and pathogenesis of SARS-CoV-2. An observational study in 54 individuals with COVID-19 found evidence of profound immune dysregulation precipitated by monocyte dysfunction in all patients (Giamarellos-Bourboulis et al. 2020). The authors noted that a sudden decrease in monocyte expression of the human leukocyte antigen (HLA)-DR immediately preceded progression to severe respiratory failure in COVID-19 patients and that patients with more mild COVID-19-associated pneumonia had monocyte HLA-DR expression closer to that of healthy controls.

Downregulation of monocyte HLA-DR is often used as a prognostic indicator of systemic inflammatory conditions including sepsis (Kim et al. 2010). HLA-DR is a major histocompatibility complex class II receptor which is responsible for antigen presentation to CD4^+^ T helper cells. Downregulation of HLA-DR on monocytes and other antigen-presenting cells may therefore suppress the transition to adaptive immune responses during an acute infection, thereby exacerbating disease. Indeed, 100% of recovered COVID-19 patients in a recent study had virus-specific CD4^+^ T cells (Grifoni et al. 2020), which is highly suggestive of a critical role of adaptive immunity in successful clearance of SARS-CoV-2. Conditions in which adaptive immunity is suppressed, including aging (Nikolich-Žugich 2018), may therefore dispose individuals to greater severity of COVID-19, and this may be at least partly predicated on defective monocyte-T cell crosstalk. Greater frequencies of exhausted T cells were shown to be associated with severe COVID-19 (Diao et al. 2020), and T cell exhaustion has been widely described in aging (Goronzy and Weyand 2017), lending further support to the potential link between monocyte dysfunction, adaptive immune system impairments, and severe COVID-19 in older adults.

Additionally, circulating IL-6 levels are negatively correlated to monocyte HLA-DR expression, and both monocytes and CD4^+^ T cells were found to be circulating sources of IL-6 in COVID-19 patients (Giamarellos-Bourboulis et al. 2020). Six of these patients were treated with tocilizumab (a monoclonal antibody against the IL-6 receptor) and displayed rapid improvements in immune function as measured by lymphocyte counts, and treatment with tocilizumab prevented reductions in HLA-DR expression in monocytes cultured in vitro with plasma isolated from COVID-19 patients. Supporting this, a small trial in two patients with severe COVID-19 used single-cell RNA sequencing to identify a unique population of monocytes which highly expressed genes including those encoding pro-inflammatory cytokines and chemokines, in addition to proteins involved in inflammasome and complement pathways (Guo et al. 2020a). Tocilizumab treatment in these patients suppressed the expression of these genes while preserving circulating levels of T and B cells, suggesting that biologic treatments aimed at blunting monocyte-mediated cytokine storm may still allow for an effective antiviral response by the adaptive immune system. More recently, tocilizumab treatment was shown to reduce systemic inflammation (as measured by C-reactive protein), increase circulating lymphocyte levels, and reduce oxygen therapy needs in a trial of 20 patients with severe or critical COVID-19 (Xu et al. 2020). However, this was an uncontrolled study where all patients were treated with tocilizumab, which is a limitation.

A substantial increase in lung macrophage prevalence has been demonstrated in bronchoalveolar lavage fluid from nine COVID-19 patients (Liao et al. 2020), and the proportion of those which were monocyte-derived *Fcn1*^*+*^ macrophages—rather than tissue-resident alveolar macrophages—progressively increased from healthy controls (*N* = 3) to patients with mild COVID-19 (*N* = 3) and to patients with severe disease (*N* = 6). This suggests that an influx of monocytes to the pulmonary spaces is a potential determinant of severe COVID-19, and Gene Ontology analysis found that these monocyte-derived macrophages were characterized by upregulated gene transcription patterns consistent with activation of multiple inflammatory pathways. Likewise, analysis of two fatal cases of COVID-19 demonstrated increased macrophage infiltration and associated fibrosis upon necropsy (Wang et al. 2020a), and a study in 14 hospitalized COVID-19 patients identified hyperactivated lung macrophages as associated with severe disease (Chua et al. 2020). Transgenic mice expressing human ACE2 (hACE2 Tg mice) additionally had accumulation of monocytes and macrophages in the lungs after SARS-CoV-2 infection (Bao et al. 2020), providing direct experimental evidence that SARS-CoV-2 precipitates monocyte influx and macrophage accumulation during active infection.

## Direct infection of myeloid cells by SARS-CoV-2

Angiotensin-converting enzyme 2 (ACE2) has been identified as the primary cellular receptor for SARS-CoV-2 (Hoffmann et al. 2020; Zhou et al. 2020b). ACE2 was previously identified as the receptor for SARS-CoV-1 (Li et al. 2003), and physiologically, its primary function is to hydrolyze angiotensin II to angiotensin (1–7). SARS-CoV-1 downregulates ACE2 expression in infected cells, contributing to more severe disease (Kuba et al. 2005). This is likely to occur in SARS-CoV-2 infection as well (Zhang et al. 2020c); supporting this, monocytes from COVID-19 patients displayed reduced ACE2 levels compared with non-infected controls (Zhang et al. 2020b). A recent comprehensive review (Verdeccia et al. 2020) makes a compelling argument that this loss of ACE2 is a proximate cause of lung inflammatory cell infiltration and ARDS and that conditions in which ACE2 is already reduced (including aging) may exacerbate the severity of COVID-19. This latter point (that ACE2 reductions in aging mediate more severe COVID-19) is potentially paradoxical, as it is feasible that downregulation of ACE2 expression would be protective against SARS-CoV-2 infection. However, to my knowledge, there is no established link between the total amount of ACE2 expressed on a given individual cell and its susceptibility to viral infection, and therefore, this paradox may be resolved (in theory) by the hypothesis that lower expression of ACE2 on aged cells still permits viral infection, but also results in increased inflammatory responses as described above. To date, this is speculative and is deserving of direct experimental investigation.

Both human monocytes and macrophages express ACE2 (Zhao et al. 2020; Zhang et al. 2020b), making them potentially susceptible to SARS-CoV-2 infection. ACE2 expression is reduced in the circulating population of monocyte/macrophages with high forward scatter described above (Zhang et al. 2020b). This may suggest direct infection of these cells by SARS-CoV-2, but this has not been evaluated. However, some evidence exists that monocytes and macrophages are directly permissive to SARS-CoV-2 infection. In hACE2 Tg mice inoculated with SARS-CoV-2, viral antigen was detected in alveolar macrophages, suggesting direct infection by the virus (Bao et al. 2020), and this has also been observed using ex vivo infections in human lung tissue (Chu et al. 2020). Likewise, human primary monocytes support viral infection in vitro (Fintelman-Rodrigues et al. 2020).

To date, it is unclear whether monocytes and macrophages are permissive for viral replication or whether SARS-CoV-2 establishes only abortive infection in these cell types. Indeed, a variety of studies demonstrated that 2003 epidemic SARS-CoV-1 could infect but could not proliferate—or could only proliferate poorly—in monocytes and macrophages (Cheung et al. 2005; Yilla et al. 2005; Chu et al. 2014; Yip et al. 2014; Zhou et al. 2014; Tynell et al. 2016). However, even in the absence of productive infection, monocytes and macrophages still represent a significant route for viral dissemination in the host outside of the pulmonary system. Supporting this, Chen et al. (Chen et al. 2020c) found macrophages in lymph nodes and spleens to be positive for SARS-CoV-2 nucleocapsid protein antigens, and this was associated with severe pathology of those organs. Additionally, macrophage infection with the 2003 epidemic SARS-CoV-1 via Fc-receptor-mediated recognition and uptake of virus-bound antibodies has been demonstrated (Yip et al. 2014; Liu et al. 2019), suggesting a potential method by which coronaviruses may establish persistent infection in seroconverted hosts or may otherwise circumvent antiviral immune responses.

## Phenotype of monocytes in aging

Aging is characterized by substantial dysregulation of a variety of cellular functions in monocytes and macrophages. Here, I will present a very brief review of aging and monocytes, as macrophages have been more widely studied, and associated findings have been recently reviewed (Albright et al. 2016; Nikolich-Žugich 2018; van Beek et al. 2019).

Perhaps the primary hallmark of monocyte aging is skewing towards higher proportions of non-classical monocytes, with reduced circulating proportions of classical monocytes (Sadeghi et al. 1999; Nyugen et al. 2010; Seidler et al. 2010; Hearps et al. 2012; Ault et al. 2018; Ong et al. 2018; Pence and Yarbro 2018). Monocytes isolated from older adults also display increased basal (i.e., unstimulated) cytokine production compared with monocytes from younger adults (Hearps et al. 2012), thereby likely contributing to chronic low-grade age-associated inflammation commonly referred to as “inflammaging” (Franceschi et al. 2018). Mechanisms for this are not entirely clear, but my laboratory and others have recently examined age-associated changes in monocyte cellular metabolism (Pence and Yarbro 2018, 2019; Yarbro and Pence 2019; Saare et al. 2020), as metabolic reprogramming of immune function is now well-established (O’Neill et al. 2016). Aged monocytes display reduced mitochondrial function (Pence and Yarbro 2018; Saare et al. 2020), which may enhance their reliance on pro-inflammatory glycolysis for ATP production (Saare et al. 2020), thereby contributing to a mild pro-inflammatory activation phenotype. Additionally, non-classical monocytes display hallmarks of cellular senescence (Ong et al. 2018), including mitochondrial dysfunction, suggesting a potential mechanistic link between aging and shifts in monocyte subset proportions.

Interestingly, although basal cytokine production appears to be upregulated in aged monocytes, my laboratory and others have also demonstrated impaired cytokine production in these cells under inflammatory stimuli such as lipopolysaccharide (McLachlan et al. 1995; Gon et al. 1996; Pence and Yarbro 2019); however, literature in this area is inconsistent (Rudd and Banerjee 1989; Mariani et al. 2002; Hearps et al. 2012). Monocytes from older adults also display impairments in phagocytosis (Hearps et al. 2012) and reduced HLA-DR expression (Seidler et al. 2010). Together, these findings suggest that aging induces a general shift to more pro-inflammatory, dysfunctional monocytes with shifts to higher proportions of CD16^+^ subpopulations. As this aging-associated phenotype is consistent with the monocyte phenotypes observed in severe COVID-19 described above, it is highly plausible that monocyte dysfunction could play a key role in increasing SARS-CoV-2 pathogenesis in older adults.

## Monocytes in aging and COVID-19

To date, information about any age-related differential effects of SARS-CoV-2 on monocytes or macrophages is sparse. Indirect conclusions may be drawn from some of the above-referenced literature, as monocyte and macrophage dysfunctions are associated with more severe disease, and more severe disease tends to occur in older adults. However, controlled experimental studies are currently lacking. A small study in older and younger rhesus macaques found no effect of age on monocyte number or percentage due to SARS-CoV-2 infection (Yu et al. 2020), suggesting that differences in total circulating monocytes may not play a role in severity of infection in the aging context. However, phenotypic and functional parameters of these cells were not evaluated.

Experimental aging studies on COVID-19 and monocytes are complicated by several factors. Most importantly, standard laboratory mice are not susceptible to viral infection (Bao et al. 2020), and HeLa cells expressing mouse ACE2 do not support viral replication (Zhou et al. 2020b). Because of this, a large number of alternative animal models are currently under exploration, including small(er) animal models such as Syrian hamsters and ferrets (Chan et al. 2020a, b; Shi et al. 2020; Sia et al. 2020). Additionally, hACE2 Tg mice are susceptible to SARS-CoV-2 (Bao et al. 2020), although this model is not yet widely available, and the overexpression of hACE2 in all organs in these mice makes them a questionable model for human pathogenesis studies. To date, model organisms susceptible to SARS-CoV-2 develop primarily mild disease, and good laboratory models of severe COVID-19 are currently unavailable. Aged animals may help to fill this void. Indeed, 14–16-month-old Balb/c mice were found to be more susceptible to SARS-CoV-1—including supporting viral replication in the lungs—compared with younger mice which do not display disease susceptibility or support productive infection (Roberts et al. 2005).

However, aging colonies for these virus-susceptible model organisms are likely to be rare, if they are available at all, and establishing these for experimental studies will take several years. Additionally, standard reagents for assessing immune function in models other than common laboratory mice and rats are not widely available, making it potentially difficult to collect insightful data in ferrets and hamsters beyond that afforded by gross pathological examination. Non-human primates such as rhesus macaques are susceptible to the virus (Yu et al. 2020), but significant expenses and long lead times associated with aging studies in these models may make anything beyond opportunistic experiments difficult to conduct in the near future. Isolated monocytes (and monocyte-derived macrophages) from older human subjects may also provide valuable information and have the advantage of being simple to work with and readily available. These cells would provide opportunity for immediate in vitro studies examining SARS-CoV-2 infection in aged monocytes and macrophages, although in vivo studies (being unethical in human subjects) would still require model organisms. Further mechanistic investigation of monocytes from COVID-19 patients is also likely to provide insight into the contributions of these cells to disease processes.

As such, concerted efforts are necessary to establish aging colonies of susceptible animals for studying SARS-CoV-2 and COVID-19 as quickly as possible. The development of mouse-adapted virus strains, as was the case for SARS-CoV-1 (Roberts et al. 2007), may also facilitate studies on viral pathogenesis in common laboratory rodent models of aging. Indeed, a mouse-adapted strain of SARS-CoV-2 has been recently described (Dinnon et al. 2020) and should be made available to the scientific community in a relatively short order. Additionally, mouse models of accelerated aging (Yousefzadeh et al. 2019) may be of value. The status of pathogenic SARS-CoV-2 as a biosafety level-3 agent means that only a relatively small number of laboratories will be able to conduct infection studies in model organisms using pathogenic wild-type virus. Therefore, substantial scientific collaboration will be necessary in order to answer important questions regarding aging, COVID-19, and immune function.

## Conclusions

The SARS-CoV-2 pandemic is still in its early phases, and our knowledge of the virus and the mechanisms by which it causes severe disease is still extraordinarily limited. Some early observations—as well as previous knowledge from epidemic SARS-CoV-1—suggest that monocytes and pulmonary monocyte–derived macrophages play an early and key role in the progression to severe COVID-19 by promoting cytokine storm, ARDS, and disseminated peripheral tissue damage. Pathological monocyte responses in COVID-19 bear some similarities to those in aging, suggesting that monocytes may be a contributor to the disproportionate severity of COVID-19 in older adults. A great deal of experimental research is needed to conclusively test this and other hypotheses; however, SARS-CoV-2 presents some complications to this, as standard laboratory rodent models are generally not susceptible to infection. Quick action and broad collaboration among the geroscience scientific community will be necessary to overcome these challenges.

## References

[CR1] Albright JM, Dunn RC, Shults JA, Boe DM, Afshar M, Kovacs EJ (2016). Advanced age alters monocyte and macrophage responses. Antioxid Redox Signal.

[CR2] Ault R, Dwivedi V, Koivisto E, Nagy J, Miller K, Nagendran K, Chalana I, Pan X, Wang SH, Turner J (2018). Altered monocyte phenotypes but not impaired peripheral T cell immunity may explain susceptibility of the elderly to develop tuberculosis. Exp Gerontol.

[CR3] Baig AM, Khaleeq A, Ali U, Syeda H (2020). Evidence of the COVID-19 virus targeting the CNS: tissue distribution, host-virus interaction, and proposed neurotropic mechanisms. ACS Chem Neurosci.

[CR4] Bao L, Deng W, Huang B, Gao H, Liu J, Ren L, et al. The pathogenicity of SARS-CoV-2 in hACE2 transgenic mice. Nature. 2020. 10.1038/s41586-020-2312-y. 10.1038/s41586-020-2312-y 32380511

[CR5] van Beek AA, Van den Bossche J, Mastroberardino PG, de Winther MPJ, Leenen PJM (2019). Metabolic alterations in aging macrophages: ingredients for inflammaging?. Trends Immunol.

[CR6] Chan JF-W, Zhang AJ, Yuan S, Poon VK-M, Chan CC-S, Lee AC-Y, et al. Simulation of the clinical and pathological manifestations of coronavirus disease 2019 (COVID-19) in golden Syrian hamster model: implications for disease pathogenesis and transmissibility. Clin Infect Dis. 2020a. 10.1093/cid/ciaa325. 10.1093/cid/ciaa325 PMC7184405 32215622

[CR7] Chan JFW, Yuan S, Kok KH, To KK-W, Chu H, Yang J, Xing F, Liu J, Yip CCY, Poon RWS, Tsoi HW, Lo SKF, Chan KH, Poon VKM, Chan WM, Ip JD, Cai JP, Cheng VCC, Chen H, Hui CKM, Yuen KY (2020). A familial cluster of pneumonia associated with the 2019 novel coronavirus indicating person-to-person transmission: a study of a family cluster. Lancet.

[CR8] Chen G, Wu D, Guo W, Cao Y, Huang D, Wang H, Wang T, Zhang X, Chen H, Yu H, Zhang X, Zhang M, Wu S, Song J, Chen T, Han M, Li S, Luo X, Zhao J, Ning Q (2020). Clinical and immunologic features in severe and moderate coronavirus disease 2019. J Clin Invest.

[CR9] Chen N, Zhou M, Dong X, Qu J, Gong F, Han Y, Qiu Y, Wang J, Liu Y, Wei Y, Xia J, Yu T, Zhang X, Zhang L (2020). Epidemiological and clinical characteristics of 99 cases of 2019 novel coronavirus pneumonia in Wuhan, China: a descriptive study. Lancet.

[CR10] Chen Y, Feng Z, Diao B, Wang R, Wang G, Wang C, Tan Y, Liu L, Wang C, Liu Y, Liu Y, Yuan Z, Ren L, Wu Y (2020c) The novel severe acute respiratory syndrome coronavirus 2 (SARS-CoV-2) directly decimates human spleens and lymph nodes. medRxiv [preprint]. 10.1101/2020.03.27.20045427.

[CR11] Cheung CY, Poon LLM, Ng IHY, Luk W, Sia S-F, Wu MHS, Chan K-H, Yuen K-Y, Gordon S, Guan Y, Peiris JSM (2005). Cytokine responses in severe acute respiratory syndrome coronavirus-infected macrophages in vitro: possible relevance to pathogenesis. J Virol.

[CR12] Chousterman BG, Swirski FK, Weber GF (2017). Cytokine storm and sepsis disease pathogenesis. Semin Immunopathol.

[CR13] Chu H, Zhou J, Ho-Yin Wong B, Li C, Cheng ZS, Lin X, Kwok-Man Poon V, Sun T, Choi-Yi Lau C, Fuk-Woo Chan J, Kai-Wang To K, Chan KH, Lu L, Zheng BJ, Yuen KY (2014). Productive replication of Middle East respiratory syndrome coronavirus in monocyte-derived dendritic cells modulates innate immune response. Virology.

[CR14] Chu H, Chan JF, Wang Y, Yuen TT, Chai Y, Hou Y, et al. Comparative replication and immune activation profiles of SARS-CoV-2 and SARS-CoV in human lungs: an ex vivo study with implications for the pathogenesis of COVID-19. Clin Infect Dis. 2020. 10.1093/cid/ciaa410/5818134. 10.1093/cid/ciaa410 PMC7184390 32270184

[CR15] Chua RL, Lukassen S, Trump S, Hennig BP, Wendisch D, Pott F, Debnath O, Thurmann L, Kurth F, Kazmierski J, Timmerman B, Twardziok S, Schneider S, Machleidt F, Muller-Redetzky H, Krannich A, Schmidt S, Balzer F, Liebig J, Loske J, Eils J, Ishaque N, von Kalle C, Hocke A, Witzenrath M, Goffinet C, Drosten C, Laudi S, Lehmann I, Conrad C, Sander L, Eils R (2020) Cross-talk between the airway epithelium and activated immune cells defines severity in COVID-19. medRxiv [preprint]. 10.1101/2020.04.29.20084327.

[CR16] Ciabattini A, Nardini C, Santoro F, Garagnani P, Franceschi C, Medaglini D (2018). Vaccination in the elderly: the challenge of immune changes with aging. Semin Immunol.

[CR17] Desai AN, Patel P (2020). Stopping the spread of COVID-19. JAMA.

[CR18] Diao B, Wang C, Tan Y, Chen X, Liu Y, Ning L, et al. Reduction and functional exhaustion of T cells in patients with coronavirus disease 2019 (COVID-19). Front Immunol. 2020;11. 10.3389/fimmu.2020.00827. 10.3389/fimmu.2020.00827 PMC7205903 32425950

[CR19] Dinnon KH, Leist SR, Schafer A, Edwards CE, Martinez DR, Montgomery SA, West A, Yount BL, Hou YJ, Adams LE, Gully KL, Brown AJ, Huang E, Bryant MD, Choong IC, Glenn JS, Gralinski LE, Sheahan TP, Baric RS (2020) A mouse-adapted SARS-CoV-2 model for the evaluation of COVID-19 medical countermeasures. bioRxiv [preprint]. 10.1101/2020.05.06.081497.

[CR20] van Doremalen N, Bushmaker T, Morris DH, Holbrook MG, Gamble A, Williamson BN, Tamin A, Harcourt JL, Thornburg NJ, Gerber SI, Lloyd-Smith JO, de Wit E, Munster VJ (2020). Aerosol and surface stability of SARS-CoV-2 as compared with SARS-CoV-1. N Engl J Med.

[CR21] Faust JS, del Rio C. Assessment of deaths from COVID-19 and from seasonal influenza. JAMA Intern Med. 2020. 10.1001/jamainternmed.2020.2306. 10.1001/jamainternmed.2020.2306 32407441

[CR22] Fintelman-Rodrigues N, Sacramento CQ, Lima CR, da Silva FS, Ferreira AC, Mattos M, de Freitas CS, Soares VC, Dias S, Temerozo JR, Miranda M, Matos AR, Bozza FA, Carels N, Alves CR, Siqueira MM, Bozza PT, Souza TML (2020) Atazanavir inhibits SARS-CoV-2 replication and pro-inflammatory cytokine production. bioRxiv [preprint]. 10.1101/2020.04.04.020925.

[CR23] Franceschi C, Garagnani P, Parini P, Giuliani C, Santoro A (2018). Inflammaging: a new immune–metabolic viewpoint for age-related diseases. Nat Rev Endocrinol.

[CR24] Ghebreyesus TA (2020) WHO Director-General’s opening remarks at the media briefing on COVID-19 - 11 March 2020. https://www.who.int/dg/speeches/detail/who-director-general-s-opening-remarks-at-the-media-briefing-on-covid-19%2D%2D-11-march-2020. Accessed 25 Apr 2020.

[CR25] Giamarellos-Bourboulis EJ, Netea MG, Rovina N, Akinosoglou K, Antoniadou A, Antonakos N, et al. Complex immune dysregulation in COVID-19 patients with severe respiratory failure. Cell Host Microbe. 2020. 10.1016/j.chom.2020.04.009. 10.1016/j.chom.2020.04.009 PMC7172841 32320677

[CR26] Gon Y, Hashimoto S, Hayashi S, Koura T, Matsumoto K, Horie T (1996). Lower serum concentrations of cytokines in elderly patients with pneumonia and the impaired production of cytokines by peripheral blood monocytes in the elderly. Clin Exp Immunol.

[CR27] Gorbalenya AE, Baker SC, Baric RS, de Groot RJ, Drosten C, Gulyaeva AA, Haagmans BL, Lauber C, Leontovich AM, Neuman BW, Penzar D, Perlman S, Poon LLM, Samborskiy DV, Sidorov IA, Sola I, Ziebuhr J (2020). The species severe acute respiratory syndrome-related coronavirus: classifying 2019-nCoV and naming it SARS-CoV-2. Nat Microbiol.

[CR28] Goronzy JJ, Weyand CM (2017). Successful and maladaptive T cell aging. Immunity.

[CR29] Grifoni A, Weiskopf D, Ramirez SI, Mateus J, Dan JM, Moderbacher CR, et al. Targets of T cell responses to SARS-CoV2 coronavirus in humans with COVID-19 disease and unexposed individuals. Cell. 2020. 10.1016/j.cell.2020.05.015. 10.1016/j.cell.2020.05.015 PMC7237901 32473127

[CR30] Guo C, Li B, Ma H, Wang X, Cai P, Yu Q, Zhu L, Jin L, Jiang C, Fang J, Liu Q, Zong D, Zhang W, Lu Y, Li K, Gao X, Fu B, Liu L, Ma X, Weng J, Wei H, Jin T, Lin J, Qu K (2020a) Tocilizumab treatment in severe COVID-19 patients attenuates the inflammatory storm incided by monocyte centric immune interactions revealed by single-cell analysis. bioRxiv [preprint]. 10.1101/2020.04.08.029769.

[CR31] Guo YR, Cao QD, Hong ZS, Tan YY, Chen SD, Jin HJ, Tan K Sen, Wang DY, Yan Y (2020b) The origin, transmission and clinical therapies on coronavirus disease 2019 (COVID-19) outbreak - an update on the status. Mil Med Res 7:11 . 10.1186/s40779-020-00240-0. 10.1186/s40779-020-00240-0 PMC7068984 32169119

[CR32] Hamers AAJ, Dinh HQ, Thomas GD, Marcovecchio P, Blatchley A, Nakao CS, Kim C, McSkimming C, Taylor AM, Nguyen AT, McNamara CA, Hedrick CC (2019). Human monocyte heterogeneity as revealed by high-dimensional mass cytometry. Arterioscler Thromb Vasc Biol.

[CR33] He X, Lau EHY, Wu P, Deng X, Wang J, Hao X, Lau YC, Wong JY, Guan Y, Tan X, Mo X, Chen Y, Liao B, Chen W, Hu F, Zhang Q, Zhong M, Wu Y, Zhao L, Zhang F, Cowling BJ, Li F, Leung GM (2020). Temporal dynamics in viral shedding and transmissibility of COVID-19. Nat Med.

[CR34] Hearps AC, Martin GE, Angelovich TA, Cheng WJ, Maisa A, Landay AL, Jaworowski A, Crowe SM (2012). Aging is associated with chronic innate immune activation and dysregulation of monocyte phenotype and function. Aging Cell.

[CR35] Hoffmann M, Kleine-Weber H, Schroeder S, Krüger N, Herrler T, Erichsen S, Schiergens TS, Herrler G, Wu NH, Nitsche A, Müller MA, Drosten C, Pöhlmann S (2020). SARS-CoV-2 cell entry depends on ACE2 and TMPRSS2 and is blocked by a clinically proven protease inhibitor. Cell..

[CR36] Huang C, Wang Y, Li X, Ren L, Zhao J, Hu Y, Zhang L, Fan G, Xu J, Gu X, Cheng Z, Yu T, Xia J, Wei Y, Wu W, Xie X, Yin W, Li H, Liu M, Xiao Y, Gao H, Guo L, Xie J, Wang G, Jiang R, Gao Z, Jin Q, Wang J, Cao B (2020) Clinical features of patients infected with 2019 novel coronavirus in Wuhan, China. Lancet 395:497–506 . 10.1016/S0140-6736(20)30183-5. 10.1016/S0140-6736(20)30183-5 PMC7159299 31986264

[CR37] Jakubzick CV, Randolph GJ, Henson PM (2017). Monocyte differentiation and antigen-presenting functions. Nat Rev Immunol.

[CR38] Kapellos TS, Bonaguro L, Gemünd I, Reusch N, Saglam A, Hinkley ER, Schultze JL (2019). Human monocyte subsets and phenotypes in major chronic inflammatory diseases. Front Immunol.

[CR39] Kim OY, Monsel A, Bertrand M, Coriat P, Cavaillon JM, Adib-Conquy M (2010). Differential down-regulation of HLA-DR on monocyte subpopulations during systemic inflammation. Crit Care.

[CR40] Kuba K, Imai Y, Rao S, Gao H, Guo F, Guan B, Huan Y, Yang P, Zhang Y, Deng W, Bao L, Zhang B, Liu G, Wang Z, Chappell M, Liu Y, Zheng D, Leibbrandt A, Wada T, Slutsky AS, Liu D, Qin C, Jiang C, Penninger JM (2005). A crucial role of angiotensin converting enzyme 2 (ACE2) in SARS coronavirus-induced lung injury. Nat Med.

[CR41] Li W, Moore MJ, Vasllieva N, Sui J, Wong SK, Berne MA, Somasundaran M, Sullivan JL, Luzuriaga K, Greeneugh TC, Choe H, Farzan M (2003). Angiotensin-converting enzyme 2 is a functional receptor for the SARS coronavirus. Nature.

[CR42] Liao M, Liu Y, Yuan J, Wen Y, Gang X, Zhao J, et al. Single-cell landscape of bronchoalveolar immune cells in patients with COVID-19. Nat Med. 2020. 10.1038/s41591-020-0901-9. 10.1038/s41591-020-0901-9 32398875

[CR43] Liu L, Wei Q, Lin Q, Fang J, Wang H, Kwok H, et al. Anti-spike IgG causes severe acute lung injury by skewing macrophage responses during acute SARS-CoV infection. JCI Insight. 2019;4. 10.1172/jci.insight.123158. 10.1172/jci.insight.123158 PMC6478436 30830861

[CR44] Mahase E (2020). Covid-19: what treatments are being investigated?. BMJ.

[CR45] Mariani E, Meneghetti A, Neri S, Ravaglia G, Forti P, Cattini L, Facchini A (2002). Chemokine production by natural killer cells from nonagenarians. Eur J Immunol.

[CR46] McLachlan JA, Serkin CD, Morrey KM, Bakouche O (1995). Antitumoral properties of aged human monocytes. J Immunol.

[CR47] Narasimhan PB, Marcovecchio P, Hamers AAJ, Hedrick CC (2019). Nonclassical monocytes in health and disease. Annu Rev Immunol.

[CR48] Nikolich-Žugich J (2018). The twilight of immunity: emerging concepts in aging of the immune system review-article. Nat Immunol.

[CR49] Nikolich-Žugich J, Knox KS, Rios CT, Natt B, Bhattacharya D, Fain MJ (2020). SARS-CoV-2 and COVID-19 in older adults: what we may expect regarding pathogenesis, immune responses, and outcomes. GeroScience..

[CR50] Nyugen J, Agrawal S, Gollapudi S, Gupta S (2010). Impaired functions of peripheral blood monocyte subpopulations in aged humans. J Clin Immunol.

[CR51] O’Neill LAJ, Kishton RJ, Rathmell J (2016). A guide to immunometabolism for immunologists. Nat Rev Immunol.

[CR52] Onder G, Rezza G, Brusaferro S. Case-fatality rate and characteristics of patients dying in relation to COVID-19 in Italy. JAMA. 2020. 10.1001/jama.2020.4683. 10.1001/jama.2020.4683 32203977

[CR53] Ong SM, Hadadi E, Dang TM, Yeap WH, Tan CTY, Ng TP, et al. The pro-inflammatory phenotype of the human non-classical monocyte subset is attributed to senescence article. Cell Death Dis. 2018:9. 10.1038/s41419-018-0327-1. 10.1038/s41419-018-0327-1 PMC5833376 29449647

[CR54] Pence BD, Yarbro JR (2018). Aging impairs mitochondrial respiratory capacity in classical monocytes. Exp Gerontol.

[CR55] Pence BD, Yarbro JR (2019). Classical monocytes maintain ex vivo glycolytic metabolism and early but not later inflammatory responses in older adults. Immun Ageing.

[CR56] Phan LT, Nguyen TV, Luong QC, Nguyen TV, Nguyen HT, Le HQ, Nguyen TT, Cao TM, Pham QD (2020). Importation and human-to-human transmission of a novel coronavirus in Vietnam. N Engl J Med.

[CR57] Pinti M, Appay V, Campisi J, Frasca D, Fülöp T, Sauce D, Larbi A, Weinberger B, Cossarizza A (2016). Aging of the immune system: focus on inflammation and vaccination. Eur J Immunol.

[CR58] Puelles VG, Lutgehetmann M, Lindenmeyer MT, Sperhake JP, Wong MN, Allweiss L, et al. Multiorgan and renal tropism of SARS-CoV-2. N Engl J Med. 2020. 10.1056/NEJMc2011400. 10.1056/NEJMc2011400 PMC7240771 32402155

[CR59] Qi J, Zhou Y, Hua J, Zhang L, Bian J, Liu B, Zhao Z, Jin S (2020) The scRNA-seq expression profiling of the receptor ACE2 and the cellular protease TMPRSS2 reveals human organs susceptible to COVID-19 infection. bioRxiv [preprint]. 10.1101/2020.04.16.045690. 10.3390/ijerph18010284 PMC7794913 33401657

[CR60] Qin C, Zhou L, Hu Z, Zhang S, Yang S, Tao Y, et al. Dysregulation of immune response in patients with COVID-19 in Wuhan, China. Clin Infect Dis. 2020. 10.1093/cid/ciaa248. 10.1093/cid/ciaa248 PMC7108125 32161940

[CR61] Roberts A, Paddock C, Vogel L, Butler E, Zaki S, Subbarao K (2005). Aged BALB/c mice as a model for increased severity of severe acute respiratory syndrome in elderly humans. J Virol.

[CR62] Roberts A, Deming D, Paddock CD, Cheng A, Yount B, Vogel L, Herman BD, Sheahan T, Heise M, Genrich GL, Zaki SR, Baric R, Subbarao K (2007). A mouse-adapted SARS-coronavirus causes disease and mortality in BALB/c mice. PLoS Pathog.

[CR63] Ronco C, Reis T (2020). Kidney involvement in COVID-19 and rationale for extracorporeal therapies. Nat Rev Nephrol.

[CR64] Ruan Q, Yang K, Wang W, Jiang L, Song J (2020). Clinical predictors of mortality due to COVID-19 based on an analysis of data of 150 patients from Wuhan, China. Intensive Care Med.

[CR65] Rudd AG, Banerjee DK (1989). Interleukin-1 production by human monocytes in ageing and disease. Age Ageing.

[CR66] Saare M, Tserel L, Haljasmägi L, Taalberg E, Peet N, Eimre M, Vetik R, Kingo K, Saks K, Tamm R, Milani L, Kisand K, Peterson P (2020). Monocytes present age-related changes in phospholipid concentration and decreased energy metabolism. Aging Cell.

[CR67] Sadeghi HM, Schnelle JF, Thomas JK, Nishanian P, Fahey JL (1999) Phenotypic and functional characteristics of circulating monocytes of elderly persons. Exp Gerontol 34:959–970 . 10.1016/S0531-5565(99)00065-0. 10.1016/s0531-5565(99)00065-0 10673149

[CR68] Sanchez-Cerrillo I, Landete P, Aldave B, Sanchez-Alonso S, Azofra AS, Marcos-Jimenez A, Avalos E, Alcaraz-Serna A, de los Santos I, Mateu-Albero T, Esparcia L, Lopez-Sanz C, Martinez-Fleta P, Gabrie L, del Campo Guerola L, Calzada MJ, Gonzalez-Alvaro I, Alfranca A, Sanchez-Madrid F, Munoz-Calleja C, Soriano JB, Ancochea J, Martin-Gayo E (2020) Differential redistribution of activated monocyte and dendritic cell subsets to the lung associates with severity of COVID-19. medRxiv [preprint]. 10.1101/2020.05.13.20100925.

[CR69] Schett G, Sticherling M, Neurath MF (2020). COVID-19: risk for cytokine targeting in chronic inflammatory diseases?. Nat Rev Immunol.

[CR70] Seidler S, Zimmermann HW, Bartneck M, Trautwein C, Tacke F (2010). Age-dependent alterations of monocyte subsets and monocyte-related chemokine pathways in healthy adults. BMC Immunol.

[CR71] Shahid Z, Kalayanamitra R, McClafferty B, Kepko D, Ramgobin D, Patel R, Aggarwal CS, Vunnam RR, Sahu N, Bhatt D, Jones K, Golamari R, Jain R (2020). COVID-19 and older adults: what we know. J Am Geriatr Soc.

[CR72] Shi J, Wen Z, Zhong G, Yang H, Wang C, Huang B, Liu R, He X, Shuai L, Sun Z, Zhao Y, Liu P, Liang L, Cui P, Wang J, Zhang X, Guan Y, Tan W, Wu G, Chen H, Bu Z (2020). Susceptibility of ferrets, cats, dogs, and other domesticated animals to SARS–coronavirus 2. Science.

[CR73] Sia SF, Yan L, Chin AWH, Fung K, Choy K, Wong AYL, et al. Pathogenesis and transmission of SARS-CoV-2 in golden hamsters. Nature. 2020. 10.1038/s41586-020-2342-5. 10.1038/s41586-020-2342-5 PMC7394720 32408338

[CR74] Teijaro JR (2017). Cytokine storms in infectious diseases. Semin Immunopathol.

[CR75] Tynell J, Westenius V, Rönkkö E, Munster VJ, Melén K, Österlund P, Julkunen I (2016). Middle East respiratory syndrome coronavirus shows poor replication but significant induction of antiviral responses in human monocyte-derived macrophages and dendritic cells. J Gen Virol.

[CR76] Verdeccia P, Cavallini C, Spanevello A, Angeli F (2020). The pivotal link between ACE2 deficiency and SARS-CoV-2 infection. Eur J Intern Med.

[CR77] Villani AC, Satija R, Reynolds G, Sarkizova S, Shekhar K, Fletcher J, Griesbeck M, Butler A, Zheng S, Lazo S, Jardine L, Dixon D, Stephenson E, Nilsson E, Grundberg I, McDonald D, Filby A, Li W, De Jager PL, Rozenblatt-Rosen O, Lane AA, Haniffa M, Regev A, Hacohen N (2017). Single-cell RNA-seq reveals new types of human blood dendritic cells, monocytes, and progenitors. Science.

[CR78] Wang C, Xie J, Zhao L, Fei X, Zhang H, Tan Y, Zhou L, Liu Z, Ren Y, Yuan L, Zhang Y, Zhang J, Liang L, Chen X, Liu X, Wang P, Han X, Weng X, Chen Y, Yu T, Zhang X, Cai J, Chen R, Shi Z, Bian X (2020a) Alveolar macrophage activation and cytokine storm in the pathogenesis of severe COVID-19. InReview [preprint] 10.21203/rs.3.rs-19346/v1.

[CR79] Wang D, Hu B, Hu C, Zhu F, Liu X, Zhang J, Wang B, Xiang H, Cheng Z, Xiong Y, Zhao Y, Li Y, Wang X, Peng Z (2020). Clinical characteristics of 138 hospitalized patients with 2019 novel coronavirus-infected pneumonia in Wuhan, China. JAMA.

[CR80] Wang W, Tang J, Wei F (2020). Updated understanding of the outbreak of 2019 novel coronavirus (2019-nCoV) in Wuhan, China. J Med Virol.

[CR81] Wolf AA, Yáñez A, Barman PK, Goodridge HS (2019). The ontogeny of monocyte subsets. Front Immunol.

[CR82] Wölfel R, Corman VM, Guggemos W, Seilmaier M, Zange S, Müller MA, Niemeyer D, Jones TC, Vollmar P, Rothe C, Hoelscher M, Bleicker T, Brünink S, Schneider J, Ehmann R, Zwirglmaier K, Drosten C, Wendtner C (2020). Virological assessment of hospitalized patients with COVID-2019. Nature..

[CR83] Worldometer (2020) COVID-19 coronavirus pandemic. https://www.worldometers.info/coronavirus/. Accessed 21 May 2020.

[CR84] Wu JT, Leung K, Bushman M, Kishore N, Niehus R, de Salazar PM, Cowling BJ, Lipsitch M, Leung GM (2020). Estimating clinical severity of COVID-19 from the transmission dynamics in Wuhan, China. Nat Med.

[CR85] Xiao F, Sun J, Xu Y, Li F, Huang X, Li H, et al. Infectious SARS-CoV-2 in feces of patient with severe COVID-19. Emerg Infect Dis. 2020;26. 10.3201/eid2608.200681. 10.3201/eid2608.200681 PMC7392466 32421494

[CR86] Xu X, Han M, Li T, Sun W, Wang D, Fu B, Zhou Y, Zheng X, Yang Y, Li X, Zhang X, Pan A, Wei H (2020). Effective treatment of severe COVID-19 patients with tocilizumab. Proc Natl Acad Sci.

[CR87] Yarbro JR, Pence BD (2019). Classical monocytes from older adults maintain capacity for metabolic compensation during glucose deprivation and lipopolysaccharide stimulation. Mech Ageing Dev.

[CR88] Yeo C, Kaushal S, Yeo D (2020) Enteric involvement of coronaviruses: is faecal–oral transmission of SARS-CoV-2 possible? Lancet Gastroenterol Hepatol 5:335–337 . 10.1016/S2468-1253(20)30048-0. 10.1016/S2468-1253(20)30048-0 PMC7130008 32087098

[CR89] Yilla M, Harcourt BH, Hickman CJ, McGrew M, Tamin A, Goldsmith CS, Bellini WJ, Anderson LJ (2005). SARS-coronavirus replication in human peripheral monocytes/macrophages. Virus Res.

[CR90] Yip MS, Leung NHL, Cheung CY, Li PH, Lee HHY, Daëron M, Peiris JSM, Bruzzone R, Jaume M (2014). Antibody-dependent infection of human macrophages by severe acute respiratory syndrome coronavirus. Virol J.

[CR91] Young BE, Ong SWX, Kalimuddin S, Low JG, Tan SY, Loh J, Ng OT, Marimuthu K, Ang LW, Mak TM, Lau SK, Anderson DE, Chan KS, Tan TY, Ng TY, Cui L, Said Z, Kurupatham L, Chen MIC, Chan M, Vasoo S, Wang LF, Tan BH, Lin RTP, Lee VJM, Leo YS, Lye DC (2020). Epidemiologic features and clinical course of patients infected with SARS-CoV-2 in Singapore. JAMA.

[CR92] Yousefzadeh MJ, Melos KI, Angelini L, Burd CE, Robbins PD, Niedernhofer LJ (2019). Mouse models of accelerated cellular senescence. Methods Mol Biol.

[CR93] Yu P, Qi F, Xu Y, Li F, Liu P, Liu J, Bao L, Deng W, Gao H, Xiang Z, Xiao C, Lv Q, Gong S, Liu J, Song Z, Qu Y, Xue J, Wei Q, Liu M, Wang G, Wang S, Yu H, Liu X, Huang B, Wang W, Zhao L, Wang H, Ye F, Zhou W, Zhen W, Han J, Wu G, Jin Q, Wang J, Tan W, Qin C (2020). Age-related rhesus macaque models of COVID-19. Anim Model Exp Med.

[CR94] Zhang C, Shi L, Wang FS (2020). Liver injury in COVID-19: management and challenges. Lancet Gastroenterol Hepatol.

[CR95] Zhang D, Gui R, Lei L, Liu H, Wang Y, Wang Y, Qian H, Dai T, Zhang T, Lai Y, Wang J, Liu Z, Chen T, He A, O’Dwyer M, Hu J (2020b) COVID-19 infection induces readily detectable morphological and inflammation-related phenotypic changes in peripheral blood monocytes, the severity of which correlate with patient outcome. medRxiv [preprint]. 10.1101/2020.03.24.20042655.

[CR96] Zhang H, Penninger JM, Li Y, Zhong N, Slutsky AS (2020). Angiotensin-converting enzyme 2 (ACE2) as a SARS-CoV-2 receptor: molecular mechanisms and potential therapeutic target. Intensive Care Med.

[CR97] Zhang W, Zhao Y, Zhang F, Wang Q, Li T, Liu Z, Wang J, Qin Y, Zhang X, Yan X, Zeng X, Zhang S (2020). The use of anti-inflammatory drugs in the treatment of people with severe coronavirus disease 2019 (COVID-19): the experience of clinical immunologists from China. Clin Immunol.

[CR98] Zhao Y, Zhao Z, Wang Y, Zhou Y, Ma Y, Zuo W (2020) Single-cell RNA expression profiling of ACE2, the receptor of SARS-CoV-2. bioRxiv [preprint]. 10.1101/2020.01.26.919985. 10.1164/rccm.202001-0179LE PMC7462411 32663409

[CR99] Zheng YY, Ma YT, Zhang JY, Xie X (2020). COVID-19 and the cardiovascular system. Nat Rev Cardiol.

[CR100] Zhou J, Chu H, Li C, Wong BHY, Cheng ZS, Poon VKM, Sun T, Lau CCY, Wong KKY, Chan JYW, Chan JFW, Chan KH, Zheng BJ, Yuen KY, To KKW (2014). Active replication of middle east respiratory syndrome coronavirus and aberrant induction of inflammatory cytokines and chemokines in human macrophages: implications for pathogenesis. J Infect Dis.

[CR101] Zhou F, Yu T, Du R, Fan G, Liu Y, Liu Z, Xiang J, Wang Y, Song B, Gu X, Guan L, Wei Y, Li H, Wu X, Xu J, Tu S, Zhang Y, Chen H, Cao B (2020). Clinical course and risk factors for mortality of adult inpatients with COVID-19 in Wuhan, China: a retrospective cohort study. Lancet.

[CR102] Zhou P, Lou YX, Wang XG, Hu B, Zhang L, Zhang W, Si HR, Zhu Y, Li B, Huang CL, Chen HD, Chen J, Luo Y, Guo H, Di JR, Liu MQ, Chen Y, Shen XR, Wang X, Zheng XS, Zhao K, Chen QJ, Deng F, Liu LL, Yan B, Zhan FX, Wang YY, Xiao GF, Shi ZL (2020). A pneumonia outbreak associated with a new coronavirus of probable bat origin. Nature.

[CR103] Zhou Y, Fu B, Zheng X, Wang D, Zhao C, Qi Y, et al. Pathogenic T cells and inflammatory monocytes incite inflammatory storm in severe COVID-19 patients. Natl Sci Rev. 2020c. 10.1093/nsr/nwaa041. 10.1093/nsr/nwaa041 PMC7108005 34676125

[CR104] Zhou Z, Ren L, Zhang L, Zhong J, Xiao Y, Jia Z, et al. Heightened innate immune responses in the respiratory tract of COVID-19 patients. Cell Host Microbe. 2020d. 10.1016/j.chom.2020.04.017. 10.1016/j.chom.2020.04.017 PMC7196896 32407669

[CR105] Ziegler-Heitbrock L, Ancuta P, Crowe S, Dalod M, Grau V, Hart DN, Leenen PJM, Liu YJ, MacPherson G, Randolph GJ, Scherberich J, Schmitz J, Shortman K, Sozzani S, Strobl H, Zembala M, Austyn JM, Lutz MB (2010). Nomenclature of monocytes and dendritic cells in blood. Blood.

